# The influence of *FADS* genetic variation and omega-3 fatty acid deficiency on cardiometabolic disease risk in a Mexican American population

**DOI:** 10.3389/fnut.2025.1538505

**Published:** 2025-03-10

**Authors:** Sarah A. Blomquist, Jil H. Albrecht, Brian Hallmark, Yann C. Klimentidis, Luis A. Garcia, Lawrence J. Mandarino, Dawn K. Coletta, Floyd H. Chilton

**Affiliations:** ^1^School of Nutritional Sciences and Wellness, University of Arizona, Tucson, AZ, United States; ^2^Department of Medicine, College of Medicine Tucson, and Asthma and Airway Diseases Research Center, University of Arizona Health Sciences, Tucson, AZ, United States; ^3^BIO5 Institute, University of Arizona, Tucson, AZ, United States; ^4^Department of Epidemiology and Biostatistics, University of Arizona, Tucson, AZ, United States; ^5^Division of Endocrinology, Department of Medicine, The University of Arizona College of Medicine, Tucson, AZ, United States; ^6^Center for Disparities in Diabetes, Obesity and Metabolism, University of Arizona Health Sciences, Tucson, AZ, United States; ^7^Department of Physiology, The University of Arizona College of Medicine, Tucson, AZ, United States; ^8^Center for Precision Nutrition and Wellness, University of Arizona, Tucson, AZ, United States; ^9^Escuela de Medicina y Ciencias de la Salud, Tecnologico de Monterrey, Monterrey, Mexico

**Keywords:** nutrigenomics, health disparities, omega-6, omega-3, lipids, nutrition, precision nutrition

## Abstract

**Background:**

Latinos, the largest racial/ethnic minority group in the United States, have high rates of cardiometabolic diseases, hypothesized due in part to genetic variation in the fatty acid desaturase (*FADS*) cluster that is associated with reduced omega-3 (n-3) highly unsaturated fatty acid (HUFA) biosynthesis. This study examined how variations in *FADS* and other HUFA pathway-related genes (*ELOVL5* and *ELOVL2*) impact cardiometabolic disease risk factors in Latinos of Mexican Ancestry (LMA).

**Results:**

This study analyzed 493 self-identified LMA from the Arizona Insulin Resistance registry (AIR) and found a marked enrichment in *FADS* alleles linked the ancestral haplotype (AH) compared to European Americans. LMA individuals with two AH alleles produced markedly lower levels of n-6 and n-3 HUFAs. However, this was more pronounced with the n-3 HUFAs, eicosapentaenoic acid (EPA) and docosahexaenoic acid (DHA), where the n-6 arachidonic acid (ARA) to EPA and DHA ratios were 30:1 and 5:1, respectively, and circulating EPA levels were reduced to <5 ng/mL. Importantly, genetic variations in both *FADS* and *ELOVL2/5* regions also were strongly associated with several cardiometabolic disease (CMD) markers, with the presence of two *FADS* AH alleles corresponding to a 45, 33, and 41% increase in fasting insulin, triglyceride levels and HOMA-IR, respectively.

**Conclusion:**

This study reveals the potential impact of genetically influenced HUFA regulation and n-3 HUFA deficiency on cardiometabolic disease risk within LMA. These insights provide a strong rationale for future studies and clinical trials that focus on n-3 HUFA supplementation to mitigate CMD disparities in LMA populations.

## 1 Introduction

The nutritional content of food in developed nations has changed dramatically over the past 75 years (1), mirroring the rise in non-communicable diseases, which account for approximately 75% of deaths in developed countries (2–4). Cardiometabolic diseases (CMD) remain the leading cause of death globally, with disparities particularly evident among various racial/ethnic minority groups in the United States (USA). Latino populations, especially Mexican Americans (MxAm), have higher rates of obesity, diabetes, hyperlipidemia, and cardiovascular diseases compared Non-Hispanic White (5–7) populations.

It is now estimated that over 70% of daily energy consumed in a modern Western diet (MWD) is from sources that were absent from our ancestors’ hunter-gatherer diet (8). A notable example of this shift is the change in dietary polyunsaturated fatty acids (PUFA) consumption, which can be traced back to recommendations from health agencies to reduce saturated fat intake in favor of PUFAs, predominantly derived from vegetable oils such as soybean, corn, and canola (9, 10). These recommendations led to a ~ 4-fold increase in the dietary consumption of the omega-6 (n-6) PUFA linoleic acid (LA) without significantly altering the consumption of dietary omega-3 (n-3) PUFAs, thereby altering the ratio of dietary n-6 to n-3 PUFAs to greater than 10:1 (1).

**Figure 1:**
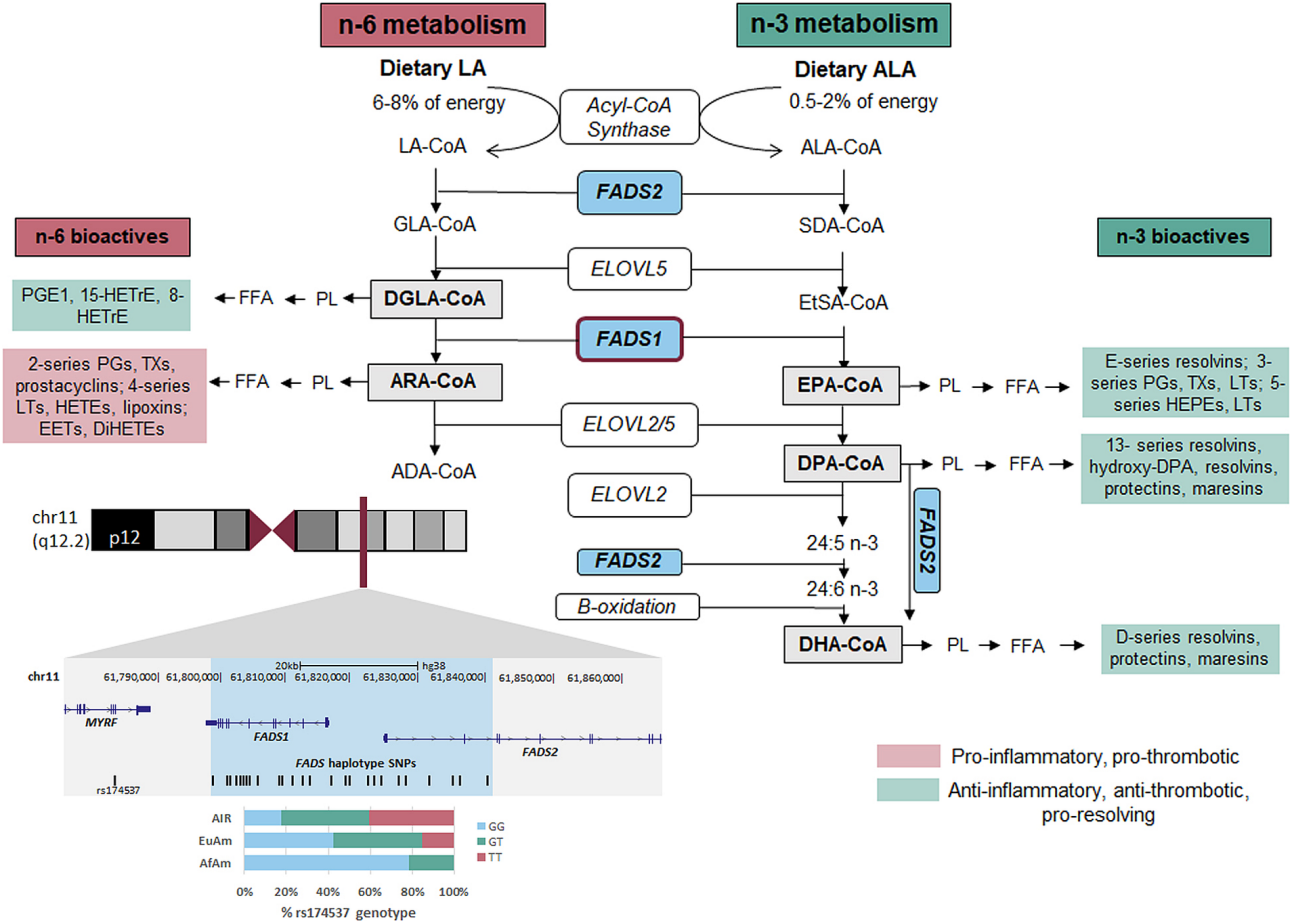
n-6 and n-3 HUFA biosynthetic pathway. *FADS1/2*, fatty acid desaturase 1 or 2; *ELOVL2/5*, fatty acid elongase 2 or 5; ALA, alpha-linolenic acid; SDA, stearidonic acid; EtSA, eicosatetranenoic acid; EPA, eicosapentaenoic acid; DPA, docosapentaenoic acid; DHA, docosahexaenoic acid; LA, linoleic acid; GLA, gamma-linolenic acid; DGLA, dihomogammalinoleic acid; ARA, arachidonic acid; ADA, adrenic acid; PL, phospholipid; FFA, free fatty acid; HETrE, hydroxyeicosatrienoic acid; PG, prostaglandin; PGE1, prostaglandin E1; TX, thromboxane; LT, leukotriene; EET, epoxyeicosatetraenoic acid; DiHETE, dihydroxyeicosatetraenoic acid; HETE, hydroxyeicosatetraenoic acid; HEPE, hydroxyeicosapentaenoic acid; Co-A, Coenzyme A.

The dietary n-6 and n-3, 18-carbon PUFAs LA and alpha-linolenic acid [ALA, omega-3 (n-3)] are converted by the liver and other tissues to long-chain (>20 carbon), highly unsaturated fatty acids (HUFAs) utilizing a sequential set of desaturases and elongases (11, 12). Figure 1 highlights genes and enzymes key to HUFA biosynthesis and metabolism. In two parallel and competing pathways, 18-carbon n-6 or n-3 PUFAs are converted into HUFAs. In the n-6 arm of the pathway (left), ARA is synthesized from LA, utilizing 2 desaturation and 1 elongation enzymatic steps (13, 14). In the n-3 arm of the pathway (right), EPA and DHA are synthesized from dietary ALA. This second desaturation step (Δ-5 desaturase or FADS1) is rate-limiting, and genetic variation throughout the *FADS* gene cluster impacts the pathway efficiency via altered *FADS1* transcription (15–17).

Once ARA is formed and placed into membrane phospholipids, it can be mobilized (by phospholipases) and metabolized [by cyclooxygenase (COX), lipoxygenase (LOX) and P450 pathways] into oxylipins including eicosanoids such as prostaglandins, thromboxanes and leukotrienes (Figure 1) (18–20). These then act locally to promote inflammation and thrombosis in CVD and other diseases (20). By contrast, DGLA, the n-6 HUFA proximal to FADS1, is metabolized by COX 1/2 to 1-series PGs, particularly PGE_1_, and by 15-LOX to 15-HETrE (21). These two metabolites of DGLA suppress inflammation, promote vasodilation, lower blood pressure, inhibit smooth muscle cell proliferation, and exert anti-neoplastic activities (22–25). Similarly, EPA and DHA are metabolized to anti-inflammatory, anti-thrombotic and “pro-resolving” oxylipins including resolvins, protectins, and maresins (19, 26, 27). With few exceptions, DGLA, EPA, DHA and their oxylipin products exert effects that differ from or oppose ARA-derived oxylipins (Figure 1) regarding inflammation, hyperlipidaemia and disease modification. Consequently, DGLA conversion to ARA and the balance of ARA to DGLA, EPA and DHA have the potential to influence the prevalence and severity of numerous human diseases.

Numerous studies have established a connection between genetic variations in the *FADS* cluster and the efficiency of the HUFA biosynthetic pathway, and thus HUFA levels (28, 29). Early research indicated that high levels of ARA, as well as the ARA/LA ratio, are independent predictors for coronary artery disease (30). Furthermore, the role of *FADS* variants in complex lipid and inflammatory phenotypes has been extensively documented (31). Notably, the *FADS* gene region is recognized as the locus associated with the largest multimorbidity cluster in the entire human genome (32).

Moreover, research from various laboratories has revealed significant ancestry-based discrepancies in the frequency of *FADS* variants affecting HUFAs and their corresponding oxylipin metabolites, such as eicosanoids (33–36). Evolutionary pressures have shaped the allele frequencies of *FADS* variants across different populations, reflecting adaptations to diverse diets and environments. For example, the “derived” *FADS* haplotype experienced ancient positive selection in Africa, leading to its high frequency in present-day African populations (37, 38). For example, ∼80% of African Americans (AfAm), as compared to ∼45% of European Americans (EuAm), carry two copies of derived alleles associated with increased *FADS1* activity, resulting in a marked increase in HUFAs, especially ARA and ARA-derived metabolites (34, 35). The introduction of agriculture is also thought to have influenced *FADS* allele frequencies. However, strong selection on the derived allele in European populations occurred much later than the Neolithic transition, possibly as late as the Bronze Age (39). The findings of Fumagalli et al. (40) on genetic adaptations in the Greenlandic Inuit population provided an important rationale as to why a high proportion of Indigenous American ancestry populations carry two copies of “ancestral” alleles associated with reduced *FADS1* activity, resulting in a reduction in HUFAs, especially EPA production (36, 37, 41, 42). Their research demonstrated that the *FADS* gene cluster shows the strongest signal of genetic adaptation, reflecting evolutionary responses to a traditional marine-based diet and extreme Arctic conditions (40). Specifically, the ancestral *FADS* alleles were positively selected in the Inuit and remain prevalent among populations with high proportions of Indigenous American ancestry such as MxAm.

An unresolved question is if the variation in *FADS* in Hispanic populations that induces very low levels of n-3 HUFAs and imbalances in n-6 to n-3 HUFA ratios influences the risk of cardiometabolic diseases often observed in these populations. To address this important question, we investigated the relationship between variation the in genes (*FADS1,2,3* and *ELOVL2,5*) that code for HUFA biosynthetic enzymes on a robust set of cardiometabolic markers and traits in adult Latino participants from the Arizona Insulin Resistance registry (AIR) (43).

## 2 Methods

### 2.1 Participants

The Arizona Insulin Resistance registry (AIR) cohort comprises 667 self-reported Latinos of Mexican Ancestry (LMA) residing in Arizona, ranging in age from 8 to 83 years. A detailed description of these participants can be found in a prior publication by Shaibi et al. (44). In the subset of *n* = 497 adult participants, 35% were male, the mean age was 36.4 and the mean BMI was 29.8. No socioeconomic status or lifestyle factors were available for reporting. Of these, 353 participants were spread across 92 families, while the remainder were single, unrelated individuals. The distribution of relative pairs within the entire cohort is documented by DeMenna et al. (43). In the current study, we focused solely on adult participants aged 18 and above (*n* = 497). In this subset, 81% were overweight or obese, and 45% had hyperglycemia (44). Comprehensive data, including metabolic, anthropometric, demographic, and medical history, were collected for all participants (44).

### 2.2 Genotyping *FADS*, *ELOVL2*, and *ELOVL5* SNPs

A total of 493 participants were successfully genotyped for 20 SNPs (three *ELOVL5*, six *ELOVL2*, and eleven *FADS1/2/3*) using the Agena Bioscience^®^ MALDI-TOF™ instrument. The 20 SNPs were chosen based on previous literature that revealed significant associations between various SNPs and HUFA levels (17, 35, 39, 40, 45–50), given that the SNPs could be successfully multiplexed. Primers for multiplex PCR and extension PCR were designed with Agena’s Online Assay Design Suite (v2.0). The assays were validated against the human genome database GRCH37/hg19 (dbSNP138). Oligos were synthesized by Integrated DNA Technologies (Coralville, Iowa), and hydrated to stock concentrations (250 μM for PCR primers and 500 μM for extension primers).

All reagents used were part of the iPlex Gold Genotyping kit (Agena Biosciences, San Diego, CA), and used according to their recommended protocols. High-quality genomic DNA was obtained from whole blood using the PAXgene Blood DNA procedure per the manufacturer’s instructions (Qiagen, CA, United States) (43). Samples were plated at 20 ng per well on a 96-well plate. A 5 μL multiplex polymerase chain reaction (PCR) was performed on each sample. Following PCR, extra nucleotides were neutralized with a shrimp alkaline phosphatase (SAP) reaction. A third reaction utilized iPLEX Gold chemistry, terminator nucleotides and a probe (extension primer) that anneals directly adjacent to the SNP site, to carry out single-base extension onto the SNP site. The reactions were cleaned using a salt-sequestering resin, and a small volume (nanoliters) of each was spotted onto the matrix of a specialized silicon chip. This chip was then analyzed using Agena’s Matrix Assisted Laser Desorption/Ionization – Time of Flight (MALDI-TOF) mass-spectrometry instrument and MassARRAY Typer software v4.1.83. Samples were genotyped using a 20-SNP custom array designed for the Agena MassARRAY MALDI-TOF instrument. The work was performed by the University of Arizona Genetics Core.

### 2.3 Description of clinical indices

Collection of samples and clinical phenotypes are described in more detail in previous publications (43, 44). Briefly, blood samples were collected after a 12-h fast to assess biomarkers including glucose, insulin, triglycerides, total cholesterol, high-density lipoprotein cholesterol, low-density lipoprotein cholesterol, and very-low-density lipoprotein cholesterol. Participants also underwent a 2-h oral glucose tolerance test. Diabetes and metabolic phenotypes, including indices of insulin action and secretion, were also collected. The following clinical indices were evaluated: body mass index (kg/m^2^), fat mass (%), waist circumference (cm), hip circumference (cm), systolic blood pressure (mmHg), diastolic blood pressure (mmHg), fasting plasma insulin (μIU/mL), fasting plasma glucose (mg/dL), hemoglobin A1c (%), 2 h oral glucose tolerance test (mg/dL), homeostasis model assessment for insulin resistance (HOMA-IR), Matsuda index, disposition index, prediabetes status, diabetes status, alanine aminotransferase (IU/L), aspartate aminotransferase (IU/L), and adiponectin (μg/mL). Participants were excluded from the study if they had any of the following: overt diabetes, untreated metabolic disease, HIV/AIDS, active cancer (or in remission for less than 3 years), acute illness, or currently pregnant.

### 2.4 Fatty acid analysis

Serum samples were collected from participants and stored at −80°C until the fatty acid (FA) analyses. Fatty acid methyl esters (FAMEs) were prepared (51) after alkaline hydrolysis of complex lipids in duplicate samples (100 μL) in the presence of the C:17 internal standard triheptadecanoin (Nuchek Prep). This standard was included for FA quantification, as previously described (52, 53). A standard panel of 37 FAs (Supelco, which accounts for 99% of FAs in the sample) was quantified by gas chromatography with flame ionization detection (GC-FID) using an Agilent 9000 Intuvo gas chromatographer with a DB-FastFAME column (20 m, 0.18 mm ID, 0.2 μm film) for a split injection with a split ratio of 15:1. The instrument response factor was calculated based on external standard sets for quality assurance purposes, and a mixture of known FAMEs was run with each sample set to monitor instrument performance (54). Individual FAs were expressed as ng/ml and were calculated using the internal standard response factor and by taking the average of duplicate samples.

### 2.5 Genetic and statistical analysis

All analyses were conducted using R Studio, version 4.2.0 or later. We examined associations between SNPs in *FADS*, *ELOVL2*, and *ELOVL5* with fatty acids (FAs) and other clinical indices using regression analyses. The major alleles found in the sampled population were chosen as the alleles whose effect was to be tested, regardless of their presence or absence in the human genome reference. Genotypes were coded based on the number of major alleles (0, 1, or 2 copies). For *FADS* SNPs, most major alleles were ancestral (i.e., the same as the most recent common ancestors of humans). The only exception was rs174538. After analyzing the 1,000 Genomes haplotype structure, it was found that the derived allele of rs174538 consistently aligns with the ancestral background. Consequently, the coding for this SNP was inverted for our analysis to again count the number of major alleles.

Linear mixed models (55) (using the lmekin package in R) with genotype coded both additively and as a factor model were performed, accounting for relatedness between individuals using self-reported pedigree information. All regression analyses included covariate adjustments for age, sex, type 2 diabetes status and body mass index (BMI). Models were also assessed after removal of BMI. Outcome variables were transformed to approximate normality using logarithmic transformations. Variables were checked for outlier data points > 3 standard deviations from the mean, but ultimately nothing was removed from the data set. Missing values were handled using listwise deletion. Summary statistic tables for all permutations of regression analysis are included in Supplementary File 1, and list *β*-values (estimate, or effect size per increasing number of major alleles), and *p*-values (significance). Due to logarithmic transformations, estimates required transformation in order to be expressed as percentages (56). Forest Plots were generated using a mixed regression model with 95% confidence intervals and are based on the standard *p*-value threshold. The false discovery rate (FDR) was controlled using the Benjamini-Hochberg procedure (57), and was performed two ways. The first set was computed from the list of all *p*-values within each individual SNP, where a threshold of 0.1 was used to examine significant associations [referred to as “pval (fdr)” in raw datasheets]. A second global FDR (*q*-values, referred to as “global_fdr” in raw datasheets) was computed using a *p*-value from all tests performed for this analysis with a 0.2 threshold for a cutoff. To account for multiple hypothesis testing and minimize false positives, we applied a false discovery rate (FDR) threshold to balance discovery and reproducibility. An FDR cutoff of 0.2, a widely used standard in genomics research (57), was initially applied to identify significant associations. To further assess the robustness of our findings, we also applied a more stringent FDR threshold of 0.1, which reduces the number of significant results by increasing conservatism. By comparing results at both thresholds, we identified the most robust associations that remained significant under the stricter criterion.

## 3 Results

### 3.1 Allele frequencies of SNPs in *FADS* and *ELOVL2/5* in different populations

Before examining allele frequencies in our study, it is important to point out that allele frequencies can vary significantly across different ethnic groups. An allele that is the major allele in one population may be the minor allele in another population. In the current study, the major alleles in Indigenous American ancestry populations (1000 Genomes MxAm and AIR) are markedly different than in AfAm and EuAm populations. As the focus of this manuscript is Indigenous American ancestry populations, the major alleles in these populations will be designated as the alleles of interest for all populations.

As discussed above and detailed in Table 1, the allele frequencies of SNPs in *FADS* and *ELOVL2/5* varied among different populations of different ancestries. For the *FADS* SNPs, the allele frequencies (based on 1,000 Genomes samples) of the alleles of interest range from 0.19 to 0.39 in EuAm populations. In AfAm populations, they range from 0.11 to 0.89, with notably smaller linkage disequilibrium (LD) blocks in the *FADS* cluster of this group, resulting in rs174594, rs174602, and rs174455 not in strong LD with other *FADS* SNPs within this population. In contrast, in 1000 Genomes MxAm populations, the frequencies were higher and ranged from 0.61 to 0.73, while in AIR, they ranged from 0.54 to 0.70.

**Table 1:** Allele frequencies for *FADS* and *ELOVL* SNPs examined in this study.

			Population allele frequencies			
	SNP	Tested allele (major in AIR)	EuAm (CEU)	AfAm (ASW)	MxAm (MXL)	AIR
*FADS*	rs174537	T	0.36	0.11	0.70	0.62
	rs174538	A	0.34	0.11	0.68	0.60
	rs174546	T	0.36	0.11	0.69	0.62
	rs174547	C	0.36	0.12	0.69	0.61
	rs174548	G	0.30	0.22	0.69	0.62
	rs174554	G	0.36	0.11	0.69	0.62
	rs1535	G	0.36	0.16	0.70	0.62
	rs174576	A	0.36	0.31	0.71	0.65
	rs174594	C	0.38	0.85	0.73	0.69
	rs174602	C	0.19	0.71	0.61	0.54
	rs174455	G	0.35	0.88	0.71	0.66
*ELOVL5*	rs7744440	T	0.74	0.35	0.77	0.71
	rs9357760	A	0.79	0.44	0.85	0.82
	rs2397142	C	0.79	0.44	0.85	0.82
*ELOVL2*	rs3734398	C	0.44	0.32	0.71	0.68
	rs2281591	A	0.76	0.53	0.80	0.74
	rs3798713	C	0.44	0.34	0.64	0.61
	rs953413	A	0.46	0.66	0.74	0.71
	rs1570069	G	0.46	0.63	0.73	0.66
	rs3798719	C	0.75	0.91	0.81	0.75

Allele frequencies were determined from 1000 Genomes populations including CEU (Utah residents with European ancestry) for EuAm, ASW (African ancestry in Southwest USA) for AfAm, and MXL (Mexican ancestry from Los Angeles, CA) for MxAm. AIR allele frequencies were calculated using data from this study. Except for rs174594, rs174602, and rs174455 in AfAm (due to smaller LD blocks in AfAm), the *FADS* SNPs were in high LD in these populations.

We also observed significant variations in genotypic frequencies across populations. As illustrated in Supplementary Figure 2, the TT genotype at rs174537 occurs in less than 1% of AfAm populations, 15% of EuAm, 48% of MxAm, and 41% of AIR participants. In contrast, the GG genotype was found in 79% AfAm, 42% EuAm, 9% MxAm and 18% AIR populations.

For the three *ELOVL5* SNPs examined, the allele frequencies in MxAm and AIR were similar to those in EuAm, ranging between 0.71 and 0.82. In contrast, in AfAm populations, they ranged from 0.35 to 0.44. Among the six *ELOVL2* SNPs, EuAm populations exhibited a frequency range from 0.44 to 0.75. AfAm populations had a broader range from 0.32 to 0.91. In MxAm and AIR populations, the frequencies lie from 0.64 to 0.81 and 0.61 to 0.75, respectively.

### 3.2 Impact of *FADS* and *ELOVL2/5* SNPs on n-6 and n-3 HUFA biosynthesis in AIR participants

**Figure 2:**
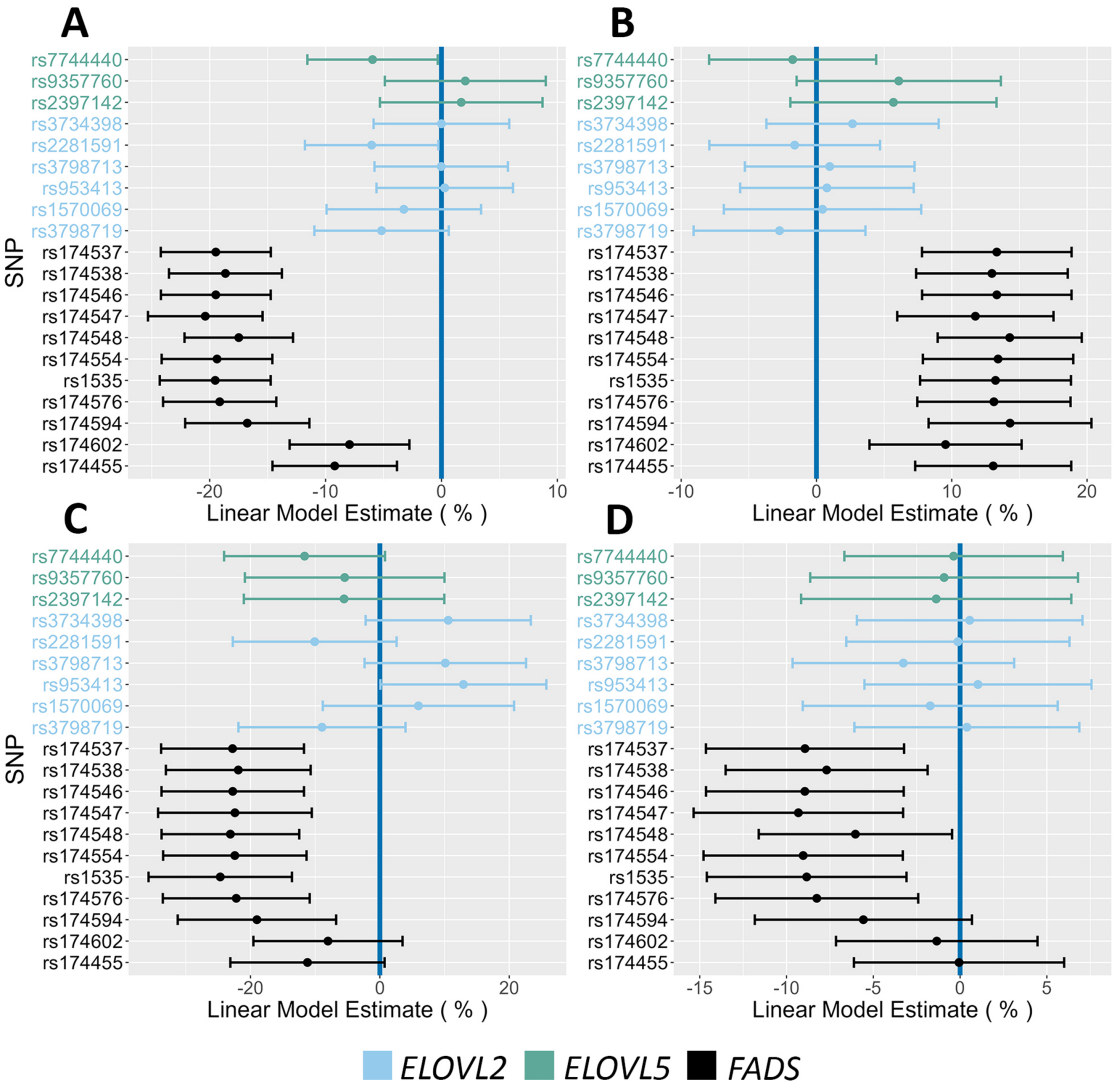
Impact of *FADS* and *ELOVL* alleles on n-6 and n-3 HUFA plasma levels. This figure contains Forest Plots that illustrate the impact of *FADS* and *ELOVL* reference alleles on n-6 and n-3 HUFA levels. **(A,B)** Allelic effects on ARA and DGLA, respectively. **(C,D)** Allelic effects on EPA and DHA, respectively. Green, blue and black lines represent *ELOVL5*, *ELOVL2* and *FADS* alleles, respectively. The 95% confidence intervals are indicated by the horizontal lines.

Figures 2A–D display Forest Plots illustrating the associations between *FADS* and *ELOVL2/5* SNP alleles and circulating n-6 and n-3 HUFA levels in the AIR cohort. All *FADS* alleles showed significant associations with the n-6 HUFAs, ARA and DGLA. For ARA levels, there was a significant decrease ranging from 8.0 to 20.4% per *FADS* major allele (the allele most common in the AIR cohort). In contrast, these same *FADS* alleles were associated with increases in DGLA levels, ranging from 9.5 to 14.3% per major allele (PMA). All but two *FADS* SNPs were associated with EPA levels, where each major allele decreased EPA levels between 11.2 and 24.7%. Similarly, all but three *FADS* SNPs were associated with DHA levels, with decreases ranging from 6.0 to 9.3% per allele. In contrast to *FADS* SNPs, only two *ELOVL2/5* SNPs had allelic associations with ARA, where each allele decreased levels by ~6% PMA. No *ELOVL2/5* alleles impacted DGLA, EPA, or DHA levels.

**Figure 3:**
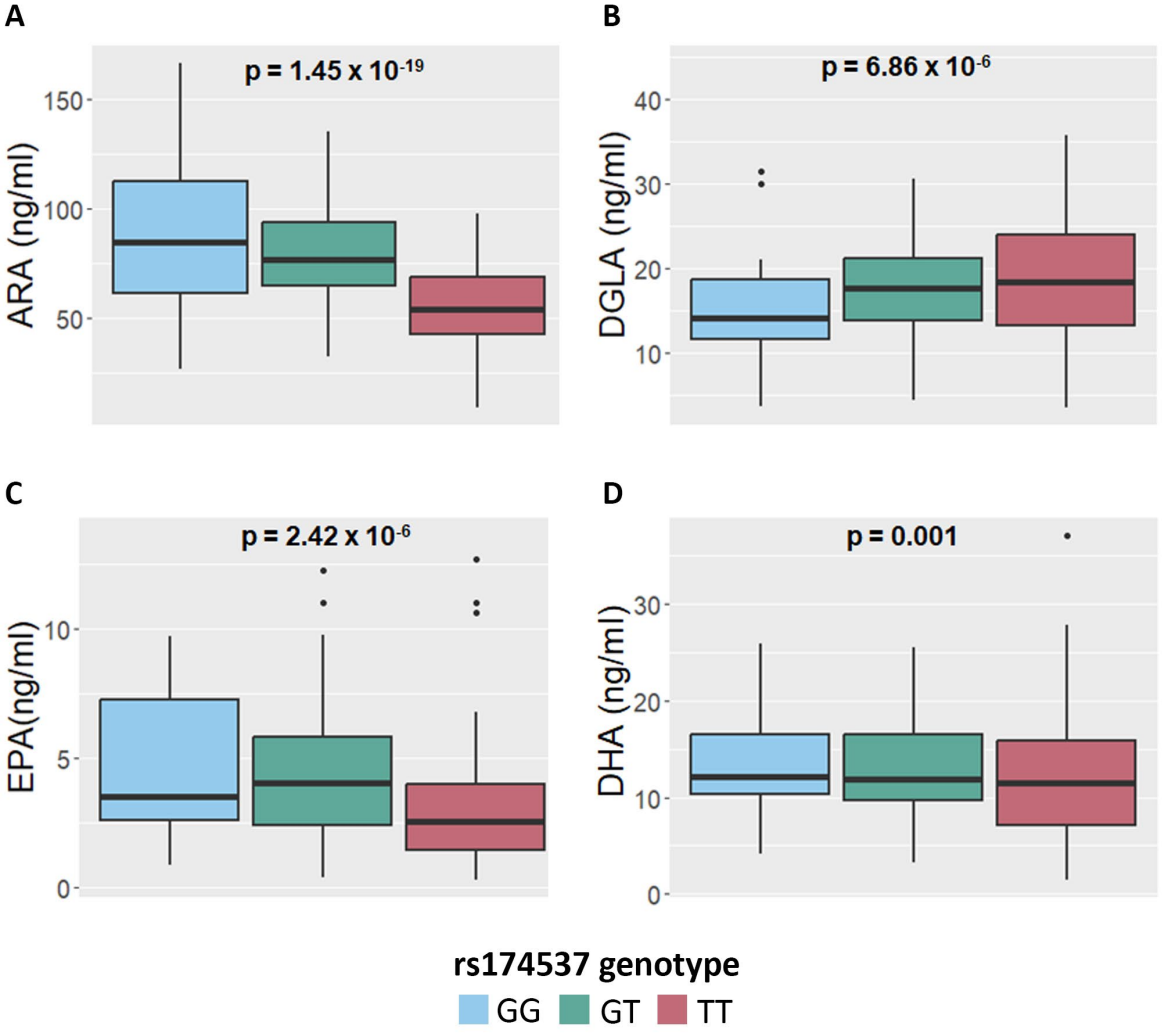
Impact of *FADS* and *ELOVL* genotypes on n-6 and n-3 HUFA plasma concentrations. **(A,B)** Concentrations of ARA and DGLA, respectively, as a function of the *FADS* SNP, rs174537, genotypes. **(C,D)** Concentrations of EPA and DHA, respectively, as a function of rs174537 genotypes. *p*-values remained significant after FDR adjustment and were derived from an additive linear mixed model.

Figures 3A–D display boxplots of HUFA levels as a function of rs174537 genotypes. Rs174537 was chosen as a representative *FADS* SNP based on its strong associations with ARA levels in previous GWAS studies, its wide use in association studies, and the fact that it sits in LD with two proposed *FADS1* functional SNPs (58, 59). Table 2 illustrates HUFA ratios, estimates (*β*) and significance values when comparing TT and GG genotypes at rs174537. Figures 2, 3 and Table 2 demonstrate a 33% reduction in ARA concentrations when comparing the GG to TT genotypes. This trend was reversed for DGLA, the HUFA proximal to the FADS1 enzymatic step, with a 32% increase in the TT compared to the GG genotype. EPA and DHA concentrations were decreased by 38 and 15% when comparing GG to TT genotypes, respectively.

**Table 2:** Impact of rs174537 genotypes on fatty acid levels.

Phenotype	Homozygous major allele (TT)	Homozygous minor allele (GG)	Estimate	95% CI	*p*-value
ARA (ng/mL)	55.83	86.23	−32.92%	(−39.10, −26.11)	3.00E-14
DGLA (ng/mL)	19.4	15.18	31.59%	(17.55, 47.31)	3.40E-05
EPA (ng/mL)	3.03	4.84	−38.30%	(−50.60, −22.94)	<0.001
DHA (ng/mL)	12.08	14.11	−15.80%	(−25.07, −5.38)	0.03
ARA/DGLA (Ratio)	3.11	6.06	−2.99	(−3.31, −2.67)	1.60E-76
ARA/EPA (Ratio)	30.53	32	−0.63	(−11.24, 9.97)	0.9
ARA/DHA (Ratio)	4.98	6.4	−1.31	(−1.69, −0.93)	8.30E-12
DHA/EPA (Ratio)	6.52	4.9	1.39	(−11.24, 9.97)	0.14

Differences in HUFA (and ratio) values when comparing the homozygous AIR population major allele (TT at rs174537) to the homozygous minor allele (GG at rs174537). The homozygous allele values were determined by calculating the mean value for rs174537 at each homozygous genotype. Estimates and unadjusted significance values were derived from summary statistics of the factor regression model. The 95% confidence interval (CI) is in the same scale as the respective estimate value.

Determining HUFA ratios throughout the biosynthesis pathway, and particularly those flanking the FADS1 biosynthetic step, has been utilized to assess the metabolic flux through the pathway (15, 16, 46). The opposing effects of n-6/n-3 HUFA on inflammatory modulation and their associated circulating levels also illustrate the balance between anti-inflammatory and pro-resolving substrates. Table 2 shows there is a 30–40% decrease in levels of both ARA and EPA individually, in individuals with the TT genotype at rs174537 (homozygous for the AIR major allele) as opposed to the GG genotype (minor in AIR but common in other populations). This is notable as these HUFAs sit at the same point (distal to the FADS1 biochemical step) in the n-6 and n-3 HUFA biosynthetic pathways.

Table 2 also reveals ARA/DGLA ratios are 3.1 and 6.1 for the TT and GG genotypes, respectively (*p* = 1.6 × 10^−76^, *β* = −2.99), suggesting that the metabolic flux through the *FADS1* portion of the pathway (Figure 1) is markedly decreased in individuals with the TT genotype. When comparing the ratios of the n-6 HUFA, ARA, to n-3 HUFAs, ARA/EPA ratios are not statistically different when comparing the TT and GG genotypes (*p* = 0.9, *β* = −0.63). In contrast, ARA/DHA ratios are statistically lower for the TT genotype (4.98 and 6.4 in the TT and GG genotypes, respectively, *p* = 8.3×10^−12^, *β* = −1.31). Within the n-3 HUFA biosynthetic pathway, DHA/EPA ratios were not significantly different when comparing TT and GG genotypes.

Supplementary Figure 3 illustrates allelic effects on HUFA ratios among all assayed SNPs. *FADS* major alleles (AIR population) were associated with decreased ratios of ARA/DGLA and ARA/DHA, where each major allele reduced the ARA/DGLA ratio by 0.75–1.5 and the ARA/DHA ratio by 0.4–0.75. *FADS* alleles at rs174548 and rs1535 also increase DHA/EPA by ~1.1. Notably, several *ELOVL2/5* alleles also impacted HUFA ratios. For example, ARA/EPA decreased by 5.7–6.4 PMA at rs3734398 and rs953413, and for ARA/DHA, rs7744440, rs2281591 and rs3798719 show a ratio decrease ranging from 0.31 to 0.33 PMA. For DHA/EPA, rs3734398, rs3798713 and rs953413 show a ratio decrease ranging from 1.2 to 1.4 PMA.

### 3.3 Association between *FADS* and *ELOVL2/5* variants and cardiometabolic biomarkers in the AIR cohort

**Figure 4:**
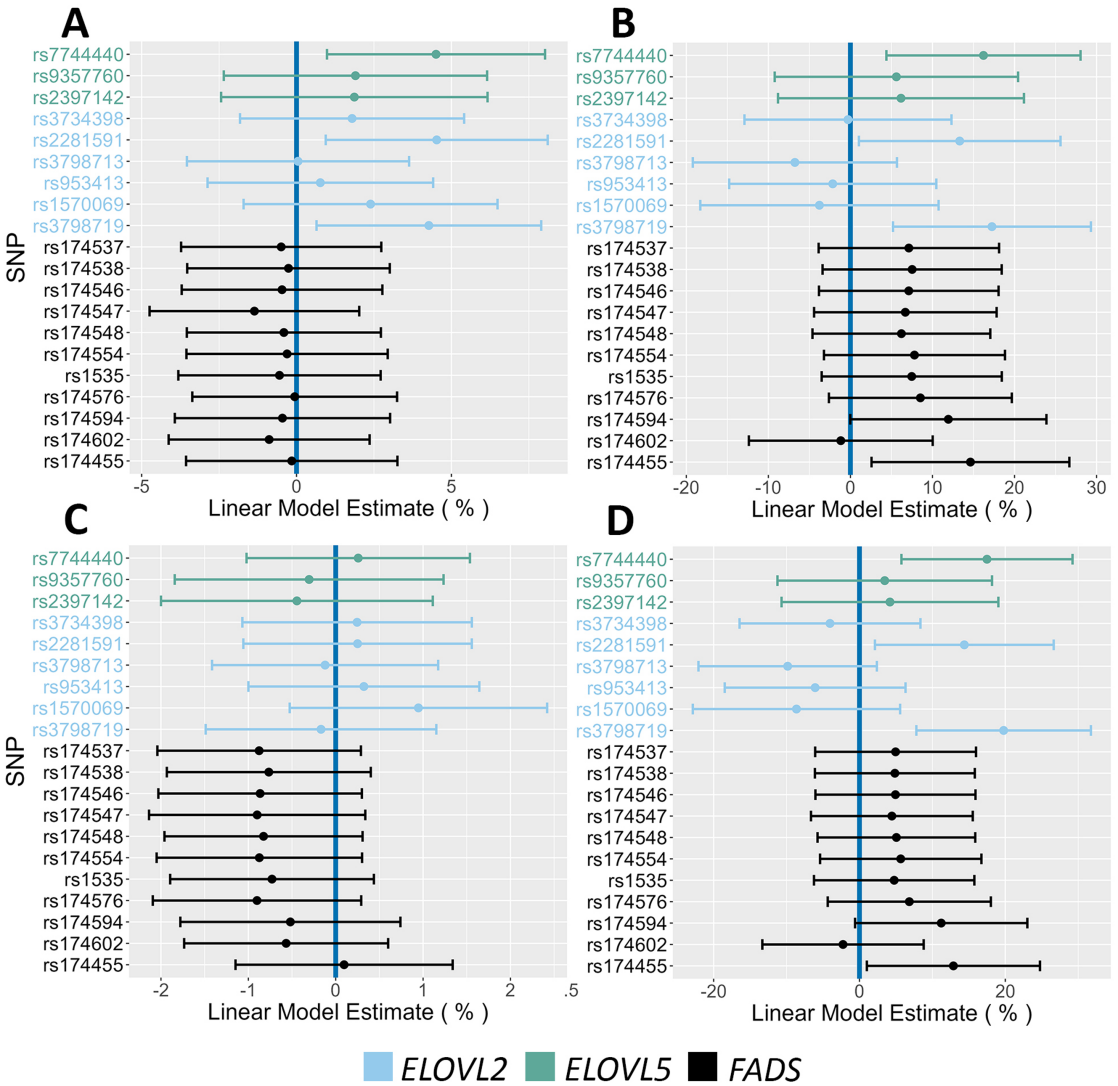
Impact of *FADS* and *ELOVL* genotypes on insulin action and glucose phenotypes. **(A,B)** Associations between 2 h glucose and HOMA-IR, respectively, and all assayed SNPs. **(C,D)** Associations between fasting glucose and fasting insulin, respectively, and all assayed SNPs. The 95% confidence intervals are indicated by the horizontal lines.

**Figure 5:**
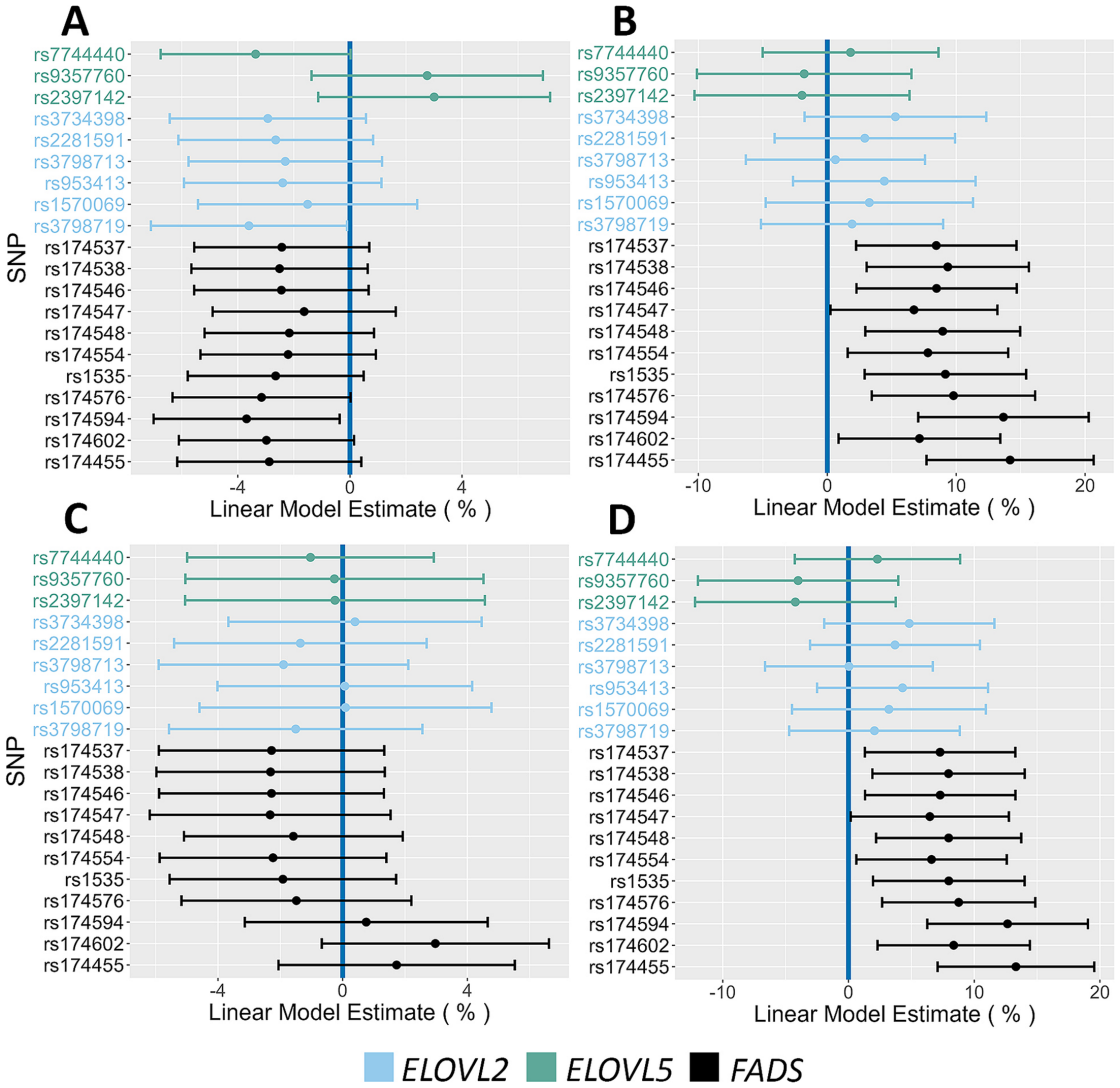
Impact of *FADS* and *ELOVL* genotypes on fasting lipid phenotypes. **(A,B)** Associations between HDL and Triglycerides, respectively, and all assayed SNPs. **(C,D)** Associations between LDL and VLDL, respectively, and all assayed SNPs. The 95% confidence intervals are indicated by the horizontal lines.

**Figure 6:**
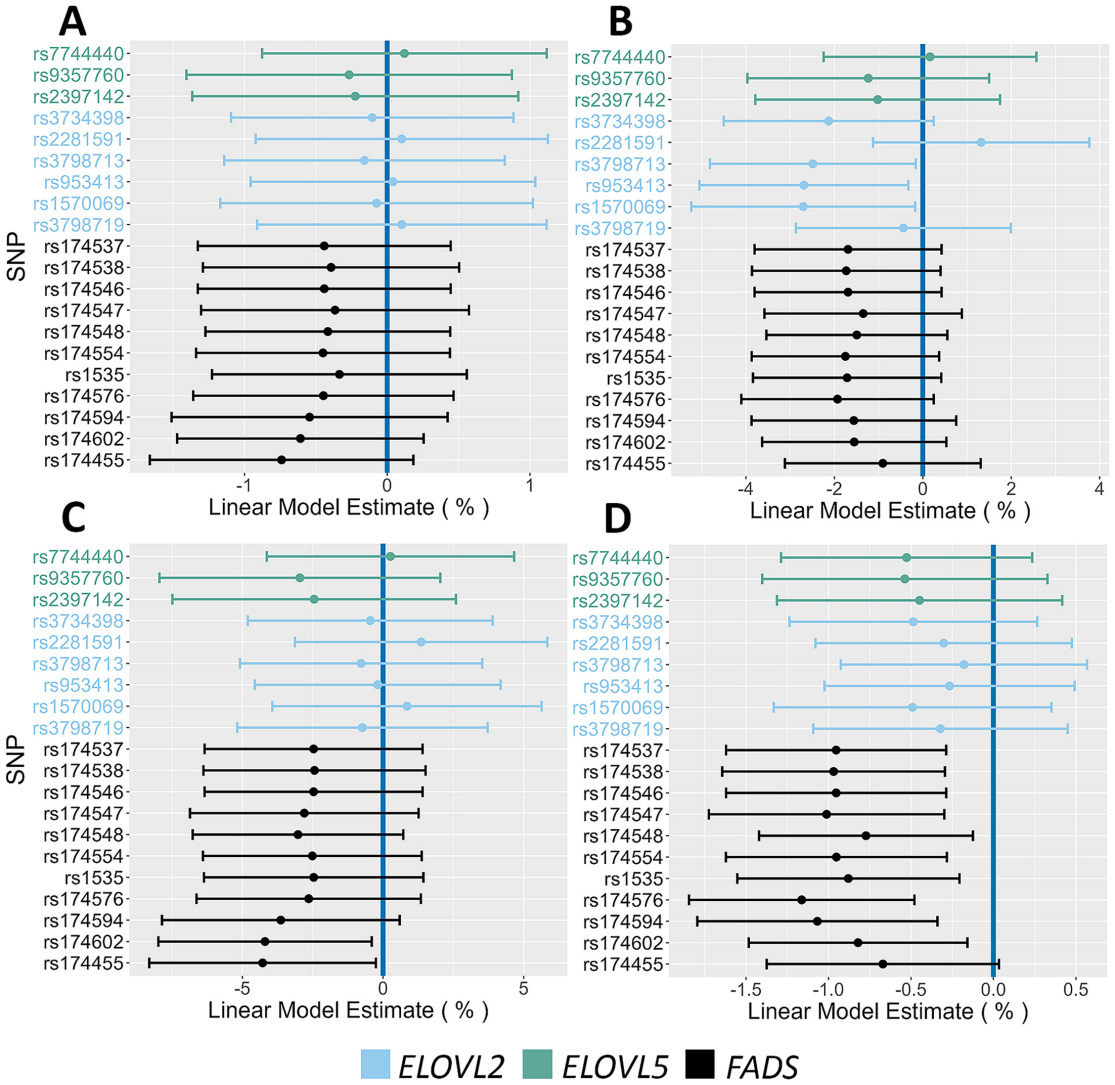
Impact of *FADS* and *ELOVL* genotypes on anthropometric phenotypes in females. **(A,B)** Associations between height and fat mass, respectively, and all assayed SNPs. **(C,D)** Associations between weight and hip circumference, respectively, and all assayed SNPs. The 95% confidence intervals are indicated by the horizontal lines.

Figure 4 displays Forest Plots that examine the associations with phenotypes linked to insulin regulation. The *FADS* SNP rs174455 was associated with both HOMA-IR (14.6% increase PMA) and fasting insulin levels (12.8% increase PMA). Three *ELOVL2/5* SNPs (rs7744440, rs2281591, rs3798719) were associated with 2 h glucose (4.3–4.5% increase PMA), fasting insulin (14.3–19.8% increase PMA), and HOMA-IR (13.3–17.2% increase PMA). Forest Plots in Figure 5 illustrate associations between the AIR cohort major alleles and fasting lipids. There were strong and consistent effects between *FADS* major alleles and increased levels of triglyceride (6.7–14.2% PMA) and VLDL (6.5–13.3% PMA). The associations of *FADS* and *ELOVL2/5* alleles with anthropometric characteristics by sex were also examined. Notably, in females, *FADS* major alleles were associated with decreased hip circumference (ranging from 0.7 to 1.2% PMA) and weight (4.1–4.2% PMA) (Figure 6). Neither *FADS* nor *ELOVL2/5* alleles had any effect on anthropomorphic traits in men (data not shown).

The *FADS* variant that showed the most consistent and strongest associations with several cardiometabolic biomarkers when comparing homozygous major alleles to homozygous minor alleles was rs174455 (Table 3). For instance, HOMA-IR values were significantly higher in the homozygous major allele genotype (GG) group compared to the homozygous minor allele genotype (AA), with a 45% increase. Similarly, fasting insulin levels were elevated by an average of 43% in individuals with the GG compared to the AA genotype. Triglyceride levels were also significantly higher in the GG group, with a 33% increase, as were VLDL levels, which rose by nearly 30%. There were no significant differences observed between GG and AA genotypes for males regarding fat mass, hip circumference, height, or weight. In females, weight was significantly different between the genotypes, with those carrying the GG genotype weighing ~10% less than their AA counterparts.

**Table 3:** Comparison of homozygous genotypes at rs174455 for cardiometabolic phenotypes.

Phenotype	Homozygous major allele (GG)	Homozygous minor allele (AA)	Estimate	95% CI	*p*-value
2 Hr Glucose (mg/dL)	138.21	130.64	−0.59%	(−7.66, 7.00)	0.87
HOMA-IR	2.31	1.78	44.96%	(11.69, 88.13)	<0.01
Fasting Glucose (mg/dL)	94.77	93.67	−0.72%	(−3.37, 2.00)	0.59
Fasting Insulin (μIU/mL/mL)	9.35	7.21	43.26%	(10.97, 84.96)	<0.01
HDL (mg/dL)	42.79	46.3	−6.06%	(−12.51, 0.85)	0.08
Triglycerides (mg/dL)	144.18	108.74	33.03%	(15.76, 52.86)	<0.01
LDL (mg/dL)	107.49	100.53	3.64%	(−4.48, 12.46)	0.39
VLDL (mg/dL)	23.8	17.29	29.90%	(13.69, 48.43)	<0.01
Male fat mass (%)	20.85	18.9	−0.44%	(−13.34, 14.36)	0.94
Female fat mass (%)	27.21	25.35	−2.46%	(−7.22, 2.54)	0.33
Male hip circumference (cm)	106.7	106.66	0.62%	(−1.79, 3.09)	0.61
Female hip circumference (cm)	110.36	109.71	−0.61%	(−2.18, 1.00)	0.46
Male height (cm)	164.82	165.56	0.26%	(2.59, 3.20)	0.85
Female height (cm)	161.85	163.13	−0.31%	(−2.38, 1.79)	0.77
Male weight (kg)	81.68	80.9	7.05%	(−4.01, 19.38)	0.22
Female weight (kg)	77.29	83.82	−9.56%	(−17.38, −1.00)	0.03

Differences in cardiometabolic phenotype values when comparing the homozygous AIR population major (GG) to the homozygous minor allele (AA) at rs174455. The homozygous allele values were compared by calculating the mean value for rs174455 for each cardiometabolic phenotype. Estimates and unadjusted significance values were derived from summary statistics of the factor regression model. The 95% confidence interval (CI) is in the same scale as the respective estimate value.

## 4 Discussion

Diabetes and hyperlipidemia are significant health concerns in Mexican and MxAm populations, who have higher prevalence rates compared to Non-Hispanic White (NHW) populations (60, 61). For example, MxAm are 1.7 times more likely to be diagnosed with diabetes than NHW, and over 50% of Hispanic adults (including MxAm) are predicted to develop type 2 diabetes in their lifetime (62). MxAm with diabetes also tend to have worse glycemic control and higher rates of diabetes-related complications, such as kidney disease. Additionally, MxAm are more likely to have higher triglyceride levels and lower HDL cholesterol levels compared to NHW (61, 62). Non-alcoholic fatty liver disease (NAFLD) affects about 25% of the general population, but the prevalence is higher in Hispanic populations, with MxAm having the highest rate at 42.8% (63).

Socioeconomic factors, including lower income, decreased access to healthcare, and language barriers, clearly contribute to the higher prevalence of these conditions in MxAm populations (61, 64). Genetic variants also play a role. For instance, the SLC16A11 risk variants, more common in people of Mexican ancestry, are associated with increased diabetes risk (64–66). Variations in the PNPLA3 gene, which predisposes people to store extra fat in liver cells, are found more often in those of Hispanic origin and are also associated with greater severity of NAFLD (67, 68).

In the current study of Latino adults of MxAm descent from the Southwest US, we observed high frequencies of alleles linked to the ancestral *FADS* haplotype, comparable to those observed in MxAm populations from the US West Coast. In contrast, these alleles are much less frequent in African and European ancestry populations and are also associated with a reduced capacity to synthesize n-6 and n-3 HUFAs, largely due to their impact on the rate-limiting *FADS1* enzymatic step (15). This observation was confirmed in our study and mirrored findings in other Latino populations (36, 41, 42), where a 33 and 38% reduction in circulating levels of ARA and EPA, respectively, was seen when individuals with the TT genotype at rs174537 were compared to those with the GG genotype.

Within the TT genotype at rs174537, mean ARA levels (56 ng/mL) were 18-fold and 5-fold higher than mean EPA (3 ng/mL) and mean DHA (12 ng/mL) levels, respectively. Moreover, individual ARA/EPA ratios display a high level of variability (coefficient of variation ~109%) with a strong positive skew, leading to a mean ARA/EPA ratio of ~30 to 1 for the TT group. Although this ratio does not differ significantly between genotypes, the extremely low concentrations of EPA in TT individuals raise an important question as to whether there is an EPA deficiency with the potential to impact cardiometabolic disease. This question is especially relevant given findings from recent notable n-3 HUFA supplementation randomized controlled trials such as ASCEND, VITAL, and REDUCE-IT (69–71). Notably, only REDUCE-IT provided higher doses of EPA to patients at high risk of CVD and demonstrated a reduction in composite CVD morbidity and mortality (including CVD mortality, non-fatal MI, non-fatal stroke, cardiovascular revascularization or unstable angina) in the treatment group (69).

While the current study corroborated the frequencies of the ancestral haplotype and its impact on n-6 and n-3 HUFA levels as has been observed in other African, European and Indigenous American ancestry cohorts, the major objective here was to determine whether *FADS*/*ELOVL* variants impact important cardiometabolic traits, including triglycerides, HDL, VLDL, fat mass, and insulin resistance markers. Previously, our laboratory examined the impact of the ancestral *FADS* haplotype on clinical phenotypes in six Hispanic subgroups in the Multi-Ethic Study of Atherosclerosis (MESA) (42). Results from this study suggested that *FADS* variation was associated with circulating levels of triglycerides, HDL-C, E-selectin, s-ICAM, waist-hip ratio, height, and weight. However, only associations for triglycerides and height remained significant when the regression model included the principal components of ancestry. That study underscored the need to investigate the clinical impact of *FADS* variation in a more homogeneous cohort.

The key findings of the current study demonstrate that the ancestral haplotype is strongly associated with several cardiometabolic endpoints. For example, at rs174455, the homozygous ancestral *FADS* genotype (compared to the homozygous derived genotype) was linked to a ~40% increase in fasting insulin and HOMA-IR, a ~30% increase in triglycerides and VLDL, and modest decreases in HDL and female anthropometric measures. Interestingly, ELOVL2/5 variants were also associated with increased glucose, insulin, and HOMA-IR, indicating variation in the HUFA biosynthetic pathway outside of the *FADS* cluster may also affect cardiometabolic endpoints. The underlying molecular mechanisms that link *FADS*, circulating HUFAs, and cardiometabolic endpoints are not yet clearly understood. However, n-3 HUFA supplementation has been shown to impact insulin-stimulated glucose disposal, potentially improving insulin sensitivity (72, 73). Additionally, the ratio of EPA to ARA has been shown to be a promising indicator of glycemic control (74).

Interestingly, alleles associated with the ancestral haplotype are often strongly linked to significantly higher triglyceride levels (36, 38, 75), whereas n-3 HUFA supplementation is well-documented to reduce circulating triglyceride levels (42, 76, 77). Mechanistically, n-3 HUFAs have been shown to: (1) inhibit hepatic synthesis and secretion of VLDL particles, the primary carriers of triglycerides in the bloodstream; (2) enhance the activity of lipoprotein lipase, an enzyme that hydrolyzes triglycerides in VLDL and chylomicrons, accelerating their clearance from circulation; (3) inhibit acyl-CoA:1,2-diacylglycerol acyltransferase, a key enzyme in triglyceride biosynthesis; and (4) increase peroxisomal *β*-oxidation, thereby promoting fatty acid metabolism (75, 76). These findings support our hypothesis that n-3 HUFA deficiency, particularly of EPA, that results from *FADS* gene-by-diet PUFA interactions in MxAm individuals of Indigenous American ancestry, may contribute to insulin resistance, elevated triglyceride levels, and an increased risk of diabetes and NAFLD. Notably, our study suggests that these metabolic conditions, when linked to n-3 HUFA deficiency, may be specifically mitigated through targeted n-3 HUFA supplementation.

A limitation of our study design is the lack of dietary data in our cohort, which prevents a complete assessment of gene-by-dietary PUFA interactions. However, we recently published data from a similar cohort of MxAm from the same region in Arizona that provide relevant context (77). In that cohort, approximately 35% of total caloric intake came from fat, with an average total fat intake of 59.6 g/day. PUFAs contributed 5–10% of energy, primarily from the n-6 PUFA, LA, with a mean intake of 12 ± 6.5 g/day (6.9 ± 2.2% of total energy). The mean n-6 to n-3 PUFA ratio in this population was 8.8:1, closely resembling the dietary patterns of other populations consuming a modern Western diet. Another limitation of our study is the lack of data on lifestyle factors and supplementation (e.g., PUFA supplements), which could influence the interpretation of our findings.

While the study’s focus on self-reported Latinos of Mexican origin from the AIR registry provided a targeted dataset, it may limit the generalizability of our findings to other Latino populations due to potential underrepresentation of broader genetic diversity. However, our analysis of the MESA cohort corroborates these findings, revealing strong negative associations between Indigenous American genetic ancestry and HUFA levels. Furthermore, the *FADS* rs174537 SNP accounted for a substantial portion of the ancestry-related effects on n-3 HUFAs, including EPA, and showed strong associations with various metabolic, inflammatory, and anthropometric traits, particularly circulating triglycerides.

Taken together, the current study suggests that gene-by-diet interactions associated with the quantities and ratios of dietary PUFAs, combined with the *FADS* ancestral haplotype, may play an important role in risk, complications and cardiometabolic disease disparities observed in MxAm populations. This study highlights the complex interplay between genetic variants, particularly in the *FADS* genes, and cardiometabolic traits in MxAm populations. The findings also underscore the importance of considering both genetic predispositions and environmental factors, such as diet, in understanding the health disparities that disproportionately affect these populations. Importantly, the gene-by-diet interactions involving PUFAs, HUFAs, and their downstream metabolic effects point to potential avenues for personalized nutrition and therapeutic strategies to mitigate cardiometabolic risk in this population.

Future research should further explore how dietary interventions, particularly those aimed at optimizing PUFA intake or supplementing with n-3 HUFAs, could modify the effects of *FADS* variants. This is particularly relevant when interactions between the *FADS* gene and dietary PUFAs result in an increased risk of n-3 HUFA deficiency, which would be expected in the 40–50% of MxAm homozygous for the ancestral haplotype. Given the high prevalence of hyperlipidemia in this population and the biological mechanisms linking n-3 HUFAs to elevated lipids, individuals in this group may especially benefit from n-3 HUFA supplementation. Supplementation with doses ranging from 2 to 4 grams per day has been shown to be most effective in lowering triglyceride levels, and the beneficial effects are typically observed within 6 weeks and can continue to improve over a period of up to 6 months in most studies (78). Additionally, expanding studies to other Latino populations and controlling for other socioeconomic and lifestyle factors will be critical in developing targeted prevention and treatment programs. Ultimately, addressing these gene-by-diet interactions could help reduce the burden of diabetes, cardiovascular disease, and related conditions in MxAm, Mexican and other Latino communities.

## Data Availability

The datasets presented in this study can be found in online repositories. The names of the repository/repositories and accession number(s) can be found in the article/Supplementary material.

## References

[1] Blasbalg TL Hibbeln JR Ramsden CE Majchrzak SF Rawlings RR. Changes in consumption of omega-3 and omega-6 fatty acids in the United States during the 20th century. Am J Clin Nutr. (2011) 93:950–62. doi: 10.3945/ajcn.110.006643, PMID: 21367944 PMC3076650

[2] Hambleton IR Caixeta R Jeyaseelan SM Luciani S Hennis AJM. The rising burden of non-communicable diseases in the Americas and the impact of population aging: a secondary analysis of available data. Lancet Reg Health Am. (2023) 21:21. doi: 10.1016/j.lana.2023.100483, PMID: 37065858 PMC10090658

[3] Richards NC Gouda HN Durham J Rampatige R Rodney A Whittaker M. Disability, noncommunicable disease and health information. Bull World Health Organ. (2016) 94:230–2. doi: 10.2471/blt.15.156869, PMID: 26966336 PMC4773929

[4] World Health Organization. Noncommunicable diseases: Mortality (2023). Available online at: https://www.who.int/data/gho/data/themes/topics/topic-details/GHO/ncd-mortality (Accessed September 26, 2024).

[5] Koyama AK McKeever Bullard K Xu F Onufrak S Jackson SL Saelee R. Prevalence of cardiometabolic diseases among racial and ethnic subgroups in adults - behavioral risk factor surveillance system, United States, 2013-2021. MMWR Morb Mortal Wkly Rep. (2024) 73:51–6. doi: 10.15585/mmwr.mm7303a1, PMID: 38271277 PMC10824545

[6] Tsao CW Aday AW Almarzooq ZI Alonso A Beaton AZ Bittencourt MS. Heart disease and stroke statistics—2022 update: a report from the American Heart Association. Circulation. (2022) 145:e153–639. doi: 10.1161/CIR.0000000000001052, PMID: 35078371

[7] Lopez-Neyman SM Davis K Zohoori N Broughton KS Moore CE Miketinas D. Racial disparities and prevalence of cardiovascular disease risk factors, cardiometabolic risk factors, and cardiovascular health metrics among us adults: NHANES 2011-2018. Sci Rep. (2022) 12:19475. doi: 10.1038/s41598-022-21878-x, PMID: 36376533 PMC9663590

[8] Cordain L Eaton SB Sebastian A Mann N Lindeberg S Watkins BA. Origins and evolution of the Western diet: health implications for the 21st century. Am J Clin Nutr. (2005) 81:341–54. doi: 10.1093/ajcn.81.2.341, PMID: 15699220

[9] Miller M Stone NJ Ballantyne C Bittner V Criqui MH Ginsberg HN. Triglycerides and cardiovascular disease: a scientific statement from the American Heart Association. Circulation. (2011) 123:2292–333. doi: 10.1161/CIR.0b013e3182160726, PMID: 21502576

[10] Dietary fat and its relation to heart attacks and strokes. Report by the Central Committee for Medical and Community Program of the American Heart Association. JAMA. (1961) 175:389–91. doi: 10.1001/jama.1961.63040050001011 14447694

[11] Brenna JT Salem N Jr Sinclair AJ Cunnane SC. Alpha-Linolenic acid supplementation and conversion to n-3 long-chain polyunsaturated fatty acids in humans. Prostaglandins Leukot Essent Fatty Acids. (2009) 80:85–91. doi: 10.1016/j.plefa.2009.01.004, PMID: 19269799

[12] Zhang JY Kothapalli KS Brenna JT. Desaturase and elongase-limiting endogenous long-chain polyunsaturated fatty acid biosynthesis. Curr Opin Clin Nutr Metab Care. (2016) 19:103–10. doi: 10.1097/mco.0000000000000254, PMID: 26828581 PMC4768719

[13] Park WJ Kothapalli KS Lawrence P Tyburczy C Brenna JT. An alternate pathway to long-chain polyunsaturates: the FADS2 gene product Delta8-desaturates 20:2n-6 and 20:3n-3. J Lipid Res. (2009) 50:1195–202. doi: 10.1194/jlr.M800630-JLR200, PMID: 19202133 PMC2681401

[14] Sprecher H Chen Q. Polyunsaturated fatty acid biosynthesis: a microsomal-peroxisomal process. Prostaglandins Leukot Essent Fatty Acids. (1999) 60:317–21. doi: 10.1016/s0952-3278(99)80006-4, PMID: 10471115

[15] Reynolds LM Dutta R Seeds MC Lake KN Hallmark B Mathias RA. FADS genetic and metabolomic analyses identify the ∆5 desaturase (FADS1) step as a critical control point in the formation of biologically important lipids. Sci Rep. (2020) 10:15873. doi: 10.1038/s41598-020-71948-1, PMID: 32985521 PMC7522985

[16] Kothapalli KSD Park HG Brenna JT. Polyunsaturated fatty acid biosynthesis pathway and genetics. implications for interindividual variability in prothrombotic, inflammatory conditions such as COVID-19. Prostaglandins Leukot Essent Fat Acids. (2020) 162:102183. doi: 10.1016/j.plefa.2020.102183, PMID: 33038834 PMC7527828

[17] Gieger C Geistlinger L Altmaier E Hrabé de Angelis M Kronenberg F Meitinger T. Genetics meets metabolomics: a genome-wide association study of metabolite profiles in human serum. PLoS Genet. (2008) 4:e1000282. doi: 10.1371/journal.pgen.1000282, PMID: 19043545 PMC2581785

[18] Haeggström JZ Funk CD. Lipoxygenase and leukotriene pathways: biochemistry, biology, and roles in disease. Chem Rev. (2011) 111:5866–98. doi: 10.1021/cr200246d, PMID: 21936577

[19] Serhan CN Chiang N. Resolution phase lipid mediators of inflammation: agonists of resolution. Curr Opin Pharmacol. (2013) 13:632–40. doi: 10.1016/j.coph.2013.05.012, PMID: 23747022 PMC3732499

[20] Smith WL. The eicosanoids and their biochemical mechanisms of action. Biochem J. (1989) 259:315–24. doi: 10.1042/bj2590315, PMID: 2655580 PMC1138513

[21] Borgeat P Hamberg M Samuelsson B. Transformation of arachidonic acid and homo-gamma-linolenic acid by rabbit polymorphonuclear leukocytes. Monohydroxy acids from novel lipoxygenases. J Biol Chem. (1976) 251:7816–20. doi: 10.1016/S0021-9258(19)57008-9, PMID: 826538

[22] Sergeant S Rahbar E Chilton FH. Gamma-linolenic acid, dihommo-gamma linolenic, eicosanoids and inflammatory processes. Eur J Pharmacol. (2016) 785:77–86. doi: 10.1016/j.ejphar.2016.04.020, PMID: 27083549 PMC4975646

[23] Fan YY Ramos KS Chapkin RS. Dietary gamma-linolenic acid modulates macrophage-vascular smooth muscle cell interactions. Evidence for a macrophage-derived soluble factor that downregulates DNA synthesis in smooth muscle cells. Arterioscler Thromb Vasc Biol. (1995) 15:1397–403. doi: 10.1161/01.atv.15.9.1397, PMID: 7670954

[24] Zurier RB. Role of prostaglandins E in inflammation and immune responses. Adv Prostaglandin Thromboxane Leukot Res. (1991) 21b:947–53. PMID: 1825439

[25] Tabolacci C Lentini A Provenzano B Gismondi A Rossi S Beninati S. Similar antineoplastic effects of nimesulide, a selective Cox-2 inhibitor, and prostaglandin E1 on B16-F10 murine melanoma cells. Melanoma Res. (2010) 20:273–9. doi: 10.1097/CMR.0b013e328339d8ac, PMID: 20404772

[26] Serhan CN Arita M Hong S Gotlinger K. Resolvins, docosatrienes, and neuroprotectins, novel omega-3-derived mediators, and their endogenous aspirin-triggered epimers. Lipids. (2004) 39:1125–32. doi: 10.1007/s11745-004-1339-7, PMID: 15726828

[27] Ariel A Serhan CN. Resolvins and protectins in the termination program of acute inflammation. Trends Immunol. (2007) 28:176–83. doi: 10.1016/j.it.2007.02.007, PMID: 17337246

[28] Schaeffer L Gohlke H Müller M Heid IM Palmer LJ Kompauer I. Common genetic variants of the FADS1 FADS2 gene cluster and their reconstructed haplotypes are associated with the fatty acid composition in phospholipids. Hum Mol Genet. (2006) 15:1745–56. doi: 10.1093/hmg/ddl117, PMID: 16670158

[29] Chilton FH Dutta R Reynolds LM Sergeant S Mathias RA Seeds MC. FADS genotypes and desaturase activity estimated by the ratio of arachidonic acid to linoleic acid are associated with inflammation and coronary artery disease. Nutrients. (2017) 9:1165. doi: 10.3390/nu9111165, PMID: 29068398 PMC5707637

[30] Martinelli N Girelli D Malerba G Guarini P Illig T Trabetti E. FADS genotypes and desaturase activity estimated by the ratio of arachidonic acid to linoleic acid are associated with inflammation and coronary artery disease. Am J Clin Nutr. (2008) 88:941–9. doi: 10.1093/ajcn/88.4.941, PMID: 18842780

[31] Koletzko B Reischl E Tanjung C Gonzalez-Casanova I Ramakrishnan U Meldrum S. FADS1 and FADS2 polymorphisms modulate fatty acid metabolism and dietary impact on health. Annu Rev Nutr. (2019) 39:21–44. doi: 10.1146/annurev-nutr-082018-124250 31433740

[32] Fadason T Schierding W Lumley T O’Sullivan JM. Chromatin interactions and expression quantitative trait loci reveal genetic drivers of multimorbidities. Nat Commun. (2018) 9:5198. doi: 10.1038/s41467-018-07692-y, PMID: 30518762 PMC6281603

[33] Hester AG Murphy RC Uhlson CJ Ivester P Lee TC Sergeant S. Relationship between a common variant in the fatty acid desaturase (FADS) cluster and eicosanoid generation in humans. J Biol Chem. (2014) 289:22482–9. doi: 10.1074/jbc.M114.579557, PMID: 24962583 PMC4139254

[34] Mathias RA Sergeant S Ruczinski I Torgerson DG Hugenschmidt CE Kubala M. The impact of FADS genetic variants on ω6 polyunsaturated fatty acid metabolism in African Americans. BMC Genet. (2011) 12:50. doi: 10.1186/1471-2156-12-50, PMID: 21599946 PMC3118962

[35] Sergeant S Hugenschmidt CE Rudock ME Ziegler JT Ivester P Ainsworth HC. Differences in arachidonic acid levels and fatty acid desaturase (FADS) gene variants in African Americans and European Americans with diabetes or the metabolic syndrome. Br J Nutr. (2012) 107:547–55. doi: 10.1017/s0007114511003230, PMID: 21733300 PMC3494092

[36] Sergeant S Keith BA Seeds MC Legins JA Young CB Vitolins MZ. Impact of FADS gene variation and dietary fatty acid exposure on biochemical and anthropomorphic phenotypes in a Hispanic/Latino cohort. Front Nutr. (2023) 10:1111624. doi: 10.3389/fnut.2023.1111624, PMID: 37215219 PMC10196633

[37] Harris DN Ruczinski I Yanek LR Becker LC Becker DM Guio H. Evolution of hominin polyunsaturated fatty acid metabolism: from Africa to the New World. Genome Biol Evol. (2019) 11:1417–30. doi: 10.1093/gbe/evz071, PMID: 30942856 PMC6514828

[38] Mathias RA Fu W Akey JM Ainsworth HC Torgerson DG Ruczinski I. Adaptive evolution of the FADS gene cluster within Africa. PLoS One. (2012) 7:e44926. doi: 10.1371/journal.pone.0044926, PMID: 23028684 PMC3446990

[39] Mathieson S Mathieson I. FADS1 and the timing of human adaptation to agriculture. Mol Biol Evol. (2018) 35:2957–70. doi: 10.1093/molbev/msy180, PMID: 30272210 PMC6278866

[40] Fumagalli M Moltke I Grarup N Racimo F Bjerregaard P Jorgensen ME. Greenlandic Inuit show genetic signatures of diet and climate adaptation. Science. (2015) 349:1343–7. doi: 10.1126/science.aab2319, PMID: 26383953

[41] Downie CG Highland HM Alotaibi M Welch BM Howard AG Cheng S. Genome-wide association study reveals shared and distinct genetic architecture underlying fatty acid and bioactive oxylipin metabolites in the Hispanic Community Health Study/Study of Latinos (HCHS/SOL). medRxiv. (2024). doi: 10.1101/2024.05.21.24307719 PMC11751521 39644095

[42] Yang C Hallmark B Chai JC O'Connor TD Reynolds LM Wood AC. Impact of Amerind ancestry and FADS genetic variation on omega-3 deficiency and cardiometabolic traits in Hispanic populations. Commun Biol. (2021) 4:918. doi: 10.1038/s42003-021-02431-4, PMID: 34321601 PMC8319323

[43] DeMenna J Puppala S Chittoor G Schneider J Kim JY Shaibi GQ. Association of common genetic variants with diabetes and metabolic syndrome related traits in the Arizona Insulin Resistance registry: a focus on Mexican American families in the Southwest. Hum Hered. (2014) 78:47–58. doi: 10.1159/000363411, PMID: 25060389 PMC4910511

[44] Shaibi GQ Coletta DK Vital V Mandarino LJ. The design and conduct of a community-based registry and biorepository: a focus on cardiometabolic health in Latinos. Clin Transl Sci. (2013) 6:429–34. doi: 10.1111/cts.12114, PMID: 24119012 PMC4225082

[45] Tanaka T Shen J Abecasis GR Kisialiou A Ordovas JM Guralnik JM. Genome-wide association study of plasma polyunsaturated fatty acids in the Inchianti study. PLoS Genet. (2009) 5:e1000338. doi: 10.1371/journal.pgen.1000338, PMID: 19148276 PMC2613033

[46] Mathias RA Vergara C Gao L Rafaels N Hand T Campbell M. FADS genetic variants and omega-6 polyunsaturated fatty acid metabolism in a homogeneous island population. J Lipid Res. (2010) 51:2766–74. doi: 10.1194/jlr.M008359, PMID: 20562440 PMC2918459

[47] Lemaitre RN Tanaka T Tang W Manichaikul A Foy M Kabagambe EK. Genetic loci associated with plasma phospholipid n-3 fatty acids: a meta-analysis of genome-wide association studies from the CHARGE Consortium. PLoS Genet. (2011) 7:e1002193. doi: 10.1371/journal.pgen.1002193, PMID: 21829377 PMC3145614

[48] Buckley MT Racimo F Allentoft ME Jensen MK Jonsson A Huang H. Selection in Europeans on fatty acid desaturases associated with dietary changes. Mol Biol Evol. (2017) 34:1307–18. doi: 10.1093/molbev/msx103, PMID: 28333262 PMC5435082

[49] Li X Gan ZW Ding Z Wu YX Chen XY Tian HM. Genetic variants in the ELOVL5 but not ELOVL2 gene associated with polyunsaturated fatty acids in Han Chinese breast milk. Biomed Environ Sci. (2017) 30:64–7. doi: 10.3967/bes2017.008 28245901

[50] Wu Y Wang Y Tian H Lu T Yu M Xu W. DHA intake interacts with ELOVL2 and ELOVL5 genetic variants to influence polyunsaturated fatty acids in human milk. J Lipid Res. (2019) 60:1043–9. doi: 10.1194/jlr.M090951, PMID: 30914501 PMC6495163

[51] Metcalfe LD Schmitz AA Pelka JR. Rapid preparation of fatty acid esters from lipids for gas chromatographic analysis. Anal Chem. (1966) 38:514–5. doi: 10.1021/ac60235a044, PMID: 39967648

[52] Sergeant S Ruczinski I Ivester P Lee TC Morgan TM Nicklas BJ. Impact of methods used to express levels of circulating fatty acids on the degree and direction of associations with blood lipids in humans. Br J Nutr. (2016) 115:251–61. doi: 10.1017/S0007114515004341, PMID: 26615716 PMC4697295

[53] Weaver KL Ivester P Seeds M Case LD Arm JP Chilton FH. Effect of dietary fatty acids on inflammatory gene expression in healthy humans. J Biol Chem. (2009) 284:15400–7. doi: 10.1074/jbc.M109.004861, PMID: 19359242 PMC2708836

[54] Brenna JT Plourde M Stark KD Jones PJ Lin YH. Best practices for the design, laboratory analysis, and reporting of trials involving fatty acids. Am J Clin Nutr. (2018) 108:211–27. doi: 10.1093/ajcn/nqy089, PMID: 29931035 PMC6084616

[55] McArdle PF O'Connell JR Pollin TI Baumgarten M Shuldiner AR Peyser PA. Accounting for relatedness in family based genetic association studies. Hum Hered. (2007) 64:234–42. doi: 10.1159/000103861, PMID: 17570925 PMC2880729

[56] Interpreting log transformations in a linear model. Available online at: https://library.virginia.edu/data/articles/interpreting-log-transformations-in-a-linear-model (Accessed October 3, 2024).

[57] Benjamini Y Drai D Elmer G Kafkafi N Golani I. Controlling the false discovery rate in behavior genetics research. Behav Brain Res. (2001) 125:279–84. doi: 10.1016/s0166-4328(01)00297-2, PMID: 11682119

[58] Hawe JS Wilson R Schmid KT Zhou L Lakshmanan LN Lehne BC. Genetic variation influencing DNA methylation provides insights into molecular mechanisms regulating genomic function. Nat Genet. (2022) 54:18–29. doi: 10.1038/s41588-021-00969-x, PMID: 34980917 PMC7617265

[59] Hermant X Delay C Flaig A Luque-Bedregal J Briand G Bout MA. Identification of a functional FADS1 3’UTR variant associated with erythrocyte n-6 polyunsaturated fatty acids levels. J Clin Lipidol. (2018) 12:1280–9. doi: 10.1016/j.jacl.2018.07.012, PMID: 30170993

[60] Statistics About Diabetes: American Diabetes Association. Available online at: https://diabetes.org/about-diabetes/statistics/about-diabetes (Accessed September 11, 2024).

[61] Aguayo-Mazzucato C Diaque P Hernandez S Rosas S Kostic A Caballero AE. Understanding the growing epidemic of type 2 diabetes in the Hispanic population living in the United States. Diabetes Metab Res Rev. (2019) 35:e3097. doi: 10.1002/dmrr.3097, PMID: 30445663 PMC6953173

[62] Vidal TM Williams CA Ramoutar UD Haffizulla F. Type 2 diabetes mellitus in Latinx populations in the United States: a culturally relevant literature review. Cureus. (2022) 14:e23173. doi: 10.7759/cureus.23173, PMID: 35444916 PMC9009996

[63] Saab S Manne V Nieto J Schwimmer JB Chalasani NP. Nonalcoholic fatty liver disease in Latinos. Clin Gastroenterol Hepatol. (2016) 14:5–12. doi: 10.1016/j.cgh.2015.05.001 25976180

[64] Fernandez ML. Lifestyle factors and genetic variants associated to health disparities in the Hispanic population. Nutrients. (2021) 13:2189. doi: 10.3390/nu13072189, PMID: 34202120 PMC8308310

[65] Mercader JM Florez JC. The genetic basis of type 2 diabetes in Hispanics and Latin Americans: challenges and opportunities. Front Public Health. (2017) 5:5. doi: 10.3389/fpubh.2017.00329, PMID: 29376044 PMC5763127

[66] Williams AL Jacobs SBR Moreno-Macías H Huerta-Chagoya A Churchhouse C Márquez-Luna C. Sequence variants in SLC16A11 are a common risk factor for type 2 diabetes in Mexico. Nature. (2014) 506:97–101. doi: 10.1038/nature12828, PMID: 24390345 PMC4127086

[67] Trépo E Romeo S Zucman-Rossi J Nahon P. PNPLA3 gene in liver diseases. J Hepatol. (2016) 65:399–412. doi: 10.1016/j.jhep.2016.03.011, PMID: 27038645

[68] Romeo S Kozlitina J Xing C Pertsemlidis A Cox D Pennacchio LA. Genetic variation in PNPLA3 confers susceptibility to nonalcoholic fatty liver disease. Nat Genet. (2008) 40:1461–5. doi: 10.1038/ng.257, PMID: 18820647 PMC2597056

[69] Bhatt DL Steg PG Miller M Brinton EA Jacobson TA Ketchum SB. Cardiovascular risk reduction with icosapent ethyl for hypertriglyceridemia. N Engl J Med. (2019) 380:11–22. doi: 10.1056/NEJMoa1812792 30415628

[70] Bowman L Mafham M Wallendszus K Stevens W Buck G Barton J. Effects of n-3 fatty acid supplements in diabetes mellitus. N Engl J Med. (2018) 379:1540–50. doi: 10.1056/NEJMoa1804989, PMID: 30146932

[71] Manson JE Cook NR Lee IM Christen W Bassuk SS Mora S. Marine n-3 fatty acids and prevention of cardiovascular disease and cancer. N Engl J Med. (2019) 380:23–32. doi: 10.1056/NEJMoa1811403, PMID: 30415637 PMC6392053

[72] Egalini F Guardamagna O Gaggero G Varaldo E Giannone B Beccuti G. The effects of omega 3 and omega 6 fatty acids on glucose metabolism: an updated review. Nutrients. (2023) 15:15 (12). doi: 10.3390/nu15122672, PMID: 37375575 PMC10301273

[73] Sinha S Haque M Lugova H Kumar S. The effect of omega-3 fatty acids on insulin resistance. Life. (2023) 13:1322. doi: 10.3390/life13061322, PMID: 37374105 PMC10305526

[74] Poreba M Rostoff P Siniarski A Mostowik M Golebiowska-Wiatrak R Nessler J. Relationship between polyunsaturated fatty acid composition in serum phospholipids, systemic low-grade inflammation, and glycemic control in patients with type 2 diabetes and atherosclerotic cardiovascular disease. Cardiovasc Diabetol. (2018) 17:29. doi: 10.1186/s12933-018-0672-5, PMID: 29452596 PMC5815243

[75] Bays HE Tighe AP Sadovsky R Davidson MH. Prescription omega-3 fatty acids and their lipid effects: physiologic mechanisms of action and clinical implications. Expert Rev Cardiovasc Ther. (2008) 6:391–409. doi: 10.1586/14779072.6.3.391, PMID: 18327998

[76] Shearer GC Savinova OV Harris WS. Fish oil -- how does it reduce plasma triglycerides? Biochim Biophys Acta. (2012) 1821:843–51. doi: 10.1016/j.bbalip.2011.10.011, PMID: 22041134 PMC3563284

[77] Lopez-Pentecost M Hallmark B Thomson CA Chilton F Garcia DO. Association between dietary fatty acid intake and liver steatosis and fibrosis in a sample of Mexican-origin Hispanic adults with overweight or obesity. Int J Environ Res Public Health. (2023) 20:3103. doi: 10.3390/ijerph20043103, PMID: 36833798 PMC9960945

[78] Wang T Zhang X Zhou N Shen Y Li B Chen BE. Association between omega-3 fatty acid intake and dyslipidemia: a continuous dose-response meta-analysis of randomized controlled trials. J Am Heart Assoc. (2023) 12:e029512. doi: 10.1161/jaha.123.029512, PMID: 37264945 PMC10381976

