# Supplementary figures and images for: The influence of FADS genetic variation and omega-3 fatty acid deficiency on cardiometabolic disease risk in a Mexican American population
Source: Front Nutr. 2025 Mar 10;12:1538505. doi: 10.3389/fnut.2025.1538505 (PMC11932658; doi:10.3389/fnut.2025.1538505)

## Slide 1
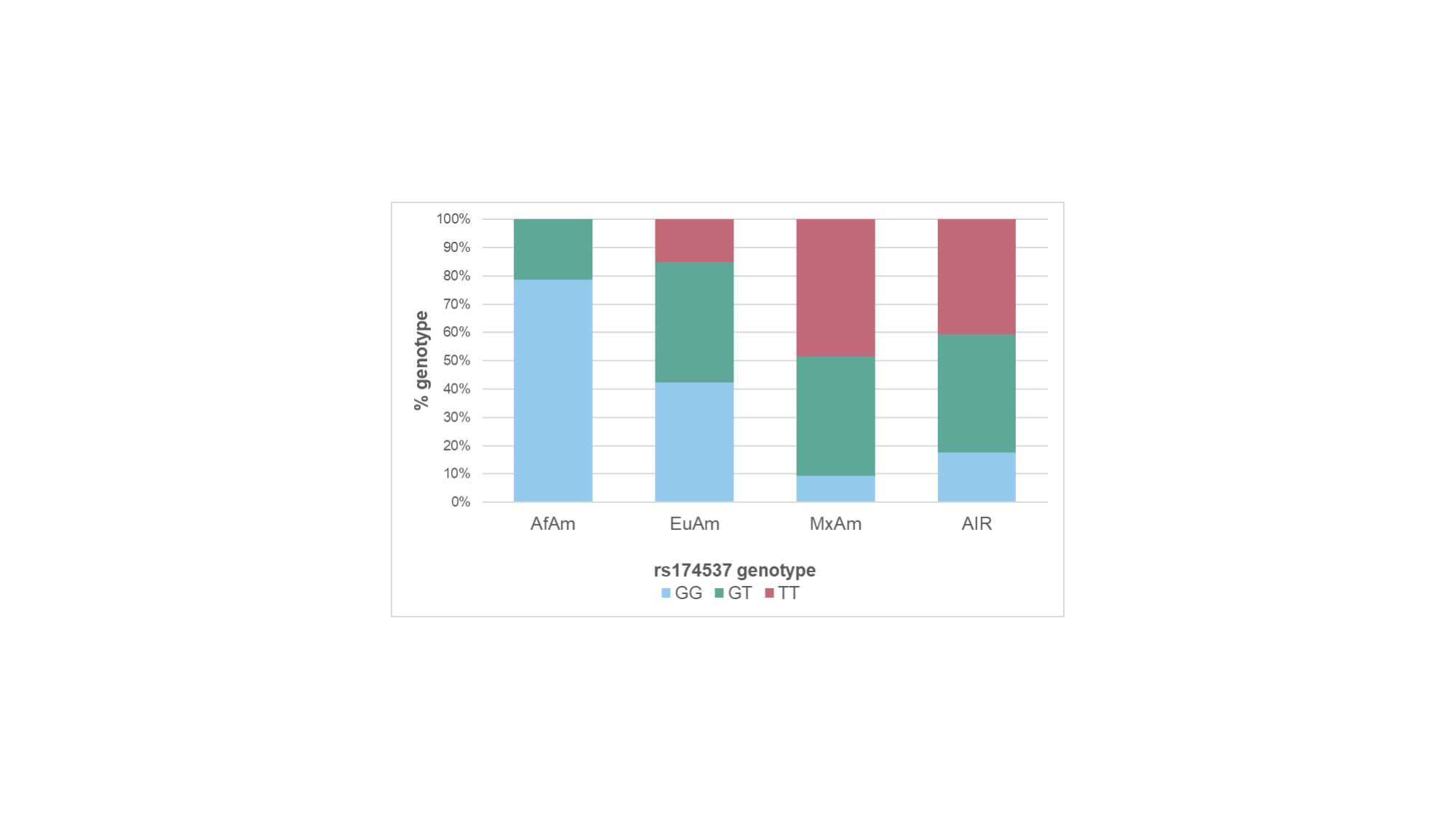

## Slide 2
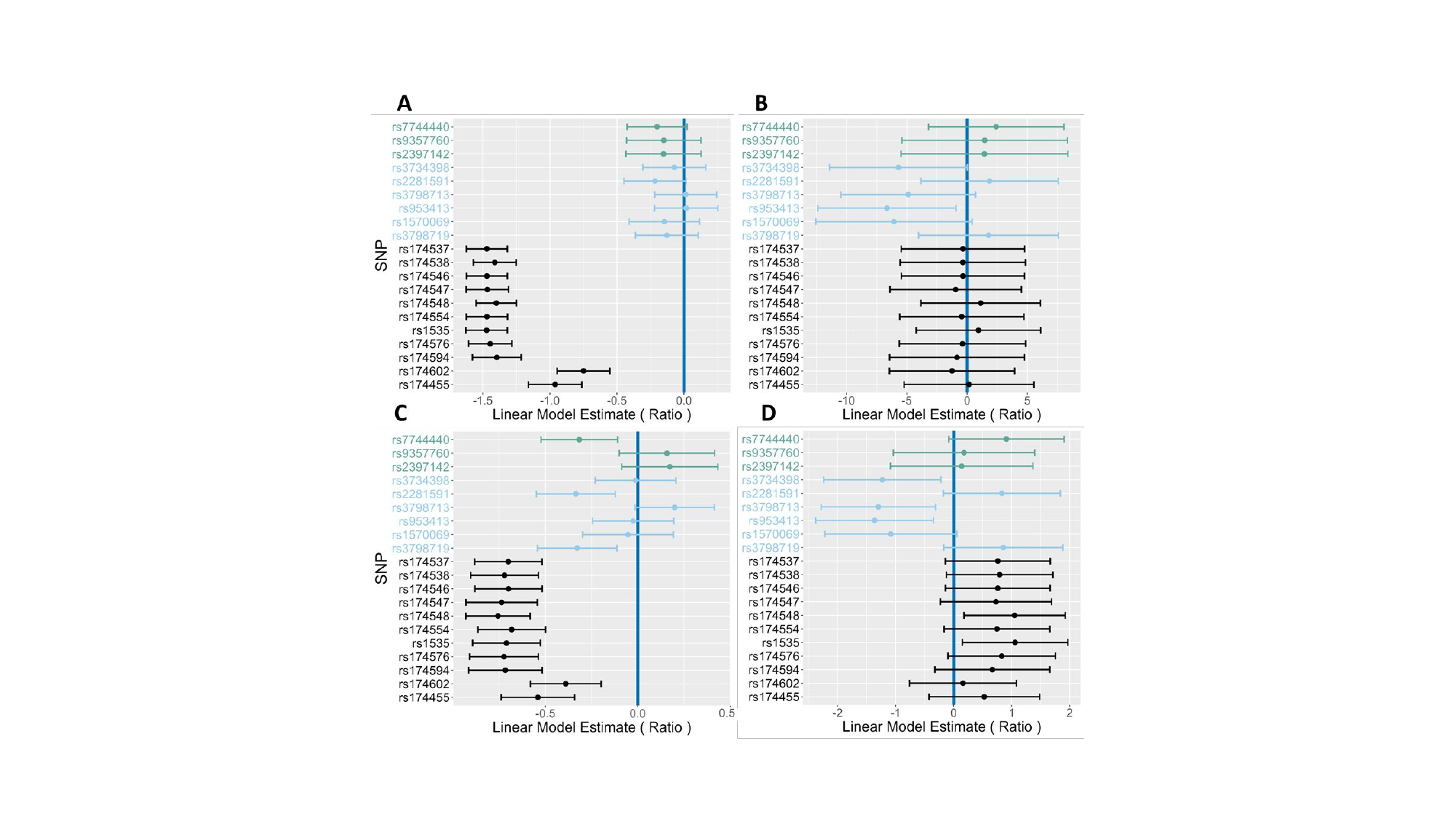

Supplement: Supplementary file 1 [file Presentation_1.pptx]
